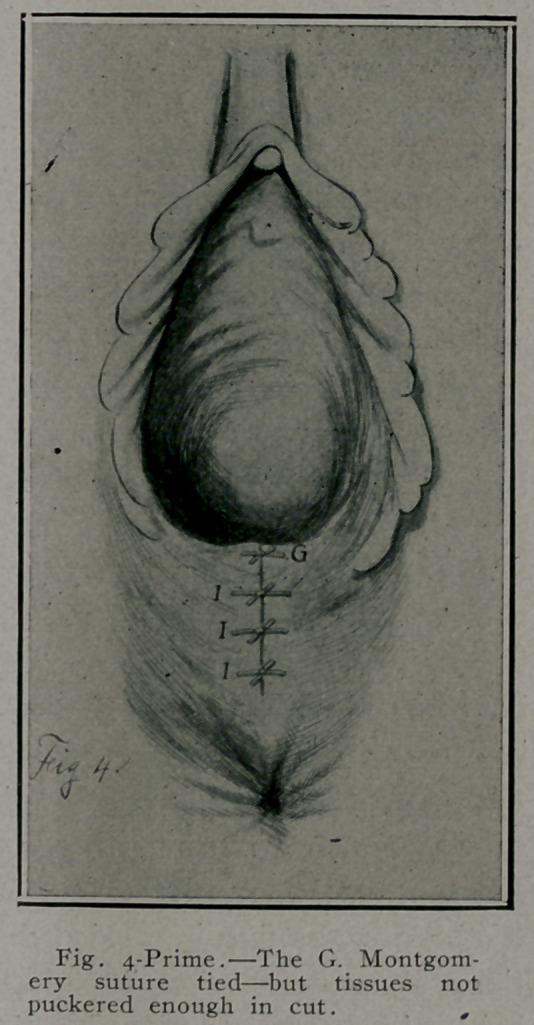# Obstetric and Gynecologic Perineal Repair

**Published:** 1911-01

**Authors:** R. R. Kime

**Affiliations:** Atlanta, Ga.


### OBSTETRIC AND GYNECOLOGIC PERINEAL REPAIR.

By R. R. Kime, M. D., Atlanta, Ga.

The same general principles apply in both obstetric and gynecological perineal repair, except in the first the surfaces are already freshened for suturing, while in the latter the surfaces have to be freshened or denuded. In the first there is no removal of tissue, so in gynecologic repair there is no need for removal of tissue in the great majority of cases.

Obstetric repair is simply bringing the parts together in their normal relation without destruction of tissue, so it should be as far as possible in gynecologic repair.

The work should be done in the simplest manner compatible with good results. The old principles enunciated by Emmett, the master of his day, hold sway in gynecology even up to the present, and in most gynecological work we have the old stereotyped cuts and teaching. The Emmett operation as originally described and performed was a complicated technique, difficult for the beginner to understand and execute and in the hands of the average operator 75 to 85% of the operations are failures in giving the patient any real benefit.

The main principles involved in the operation or any other successful operation on the perineum is the restoration of the fascical and muscular structures of the perineum. This is very easily done in obstetric repair of fresh lacerations. All one needs is a medium-sized, full-curved needle with No. 2 plain cat-gut, needle-holder and scissors to cut ligature. Buried sutures bringing together the muscles and fascia in normal relation is the essential factor which is best done by the interrupted No. 2 cat-gut suture or can be done, if preferred, by continuous buried cat-gut suture.

If the buried sutures are properly introduced, the mucous membrane and skin fall together and can be closed by a continuous over and over No. 2 cat-gut without tension. If for any reason one fears the cat-gut will absorb too soon, or in extensive lacerations, two or three interrupted sutures, No. 2 cat-gut, strand left double, can be introduced in perineal body on skin surface as stay sutures, but they are rarely needed. By this method all tissues are properly approximated, very little cutting of tissues by sutures, very little pain to patient, parts easily kept cleansed by disinfectant solution, no sutures to remove, union more perfect, and no need for silk or silk-worm gut stitches to irritate patient, to cut tissues, to hang to the dressings, being difficult to cleanse and requiring removal later.

Fig. 1.—Outline of incision for elevation of flap.

Fig. 2.—Elevation of flap complete. Sutures in left sulcus and denuded tissues introduced.

The same principles should apply to gynecologic repair, the only difference being that the submucous tissues must be exposed and brought together by proper suturing. To do this, in the great majority of cases, destruction or removal of tissues is unnecessary and unsurgical. Conservation of tissues should be one of the basic principles of surgery, and perineal repair should be no exception to that rule.

Cosmetic operations and hair-splitting dissections to expose individual muscles, except the ends of the sphincter ani when lacerated, are unnecessary and should be practised only by the expert gynecologist and not the average operator. All that is essential and necessary is to elevate the mucous membrane, exposing the tissues in the line of their original lacerations, and to bring the tissues together in their normal relation by buried catgut sutures, utilizing the mucous flap as a covering for the field of operation removing indurated scar tissue. The only ex-

Fig. 3.—Sutures tied and Montgomery stitch in flap G. introduced.

Fig. 3-Prime.—Extra sutures in perineal tissues and flap introduced.

posed sutures are in the skin surface of the perineum and edge of the mucous flap. By modifying the incision and proper elevation of the mucous flap, the essential principles in the Emmet, Hegar, Tait, or Restine operations can be carried out even in complete laceration by a very simple technique, without destruction or removal of tissue. I have advocated this method for fifteen or twenty years, presenting a paper on the subject of the Tri-State Medical Society in Nashville, Feb., 1896, from which I quote the following:

"While I do not wish to detract in the least from the Emmett operation and the fame of the master who devised it, yet I believe there are injuries to the perineum that can be repaired as accurately, effectually and scientifically by the dividing or dissecting method without the removal of tissue as by any denuding process.

Fig. 4.—Skin sutures with over-and-over suture in remains of flap shown tied.

Fig. 4-Prime.—The G. Montgomery suture tied—but tissues not puckered enough in cut.

"It matters not whether the laceration be median or lateral, the tissues can be lifted up on either side, in either sulcus, or higher in one than in the other and approximated by proper suturing without removal of the flap."

Within the last two years I have seen Drs. Murphy, Ochsner, and the Mayos do practically the same operation, while Montgomery, Watkins and others are doing similar operations, and Haynes, Amer. Jour. Obs. Dec. 1908, P. 995, has demonstrated its anatomical basis.

Years ago buried sutures in the perineum was a very difficult question compared to the present time. Some operators are yet using the chromic cat-gut. I have practically discarded it as a buried suture in the perineum unless it be in bringing the spinchter ani muscle together. In the past I have had some unpleasant experiences by the buried chromic cat-gut not absorbing, so I now prefer the plain No. 2 dropped in pure alcohol or a Tr. Iodine alcohol and water mixture before using. If there is tension on the parts or for any reason I fear too rapid absorption of the plain buried cat-gut, I use the No. 2 plain double or No. 2 chromic through the skin and perineal body, tying it externally so it can be removed if necessary. This acts as a stay suture, supports parts where there is tension, and does not cut tissue so badly as silk or silk-worm gut sutures.

The technique of the operation is simple and very much easier executed than any denuding process. Commence incision at A, Fig. 1, and extend to B, then to C along muco-cutaneous border, the incision extending up on each side to the caruncla myrtiformes. The incision is best made with scissors. The center of the flap is caught with a pair of forceps, dissected up sufficient to catch hold of it with thumb and fingers of left hand, flap being covered with gauze so as to make it easy to hold. Then the flap is easily separated with first two fingers of right hand covered with gauze, by pushing the tissues away from under surface of the mucous flap, cutting any bands of tissue as needed to expose muscles and lacerated tissue. The separation or elevation of the mucous flap is carried as high as necessary to meet indications in each individual case. If laceration extends high up in left sulcus, elevate the flap in that direction high enough to reach and suture the lacerated muscles and fascia on that side by interrupted buried No. 2 plain cat-gut sutures. Fig. 2, E. E. E. If preferred a continuous over and over suture may be used. If right sulcus is lacerated, the same procedure is carried out as on the left side. In this manner we have the Emmett principle carried out by buried sutures under the mucous flap, which serves as a protection and covering for the sutures.

After this work and in cases where laceration does not extend high in either sulcus, we have the simpler operation where the tissues are brought together in the median line. Fig. 2, F. F. F. Sutures tied in Fig. 3 and second row sutures shown H. H. H., Fig. 3-prime. In this operation there is no need of a crown stitch as in the Emmett operation; neither is there need of deep perineal stitches introduced through skin and whole perineal body. In rare cases where there is marked tension, then two or three tension sutures of cat-gut No. 2 plain double are used, or No. 2 chromic may be substituted, if preferred, and introduced just deep enough to relieve and approximate the skin.

Fig. 3 and 3-prime illustrate the approximation of the transversus perinei, anterior fibers of levator ani, pelvic fascia, and connective tissue by interrupted buried sutures under the vaginal flap. G represents a purse-string suture, as used by Montgomery, in edge of elevated vaginal flap, when tied puckers or draws in vaginal flap up to the vaginal introitus, Fig. 4-prime, G, and obviates the necessity of the over and over suture in Fig. 4.

In suturing a full-curved, medium-sized needle is used, being certain to reach well out on the side of the vagina, so as to catch up a good bite of the levator ani muscle and fascia. At times, it will be necessary to catch up anterior part of levator ani with double tinaculum and lift it up so as to include it in the suture, then come across in front of the recto-vaginal septum to the other side, getting a similar bite in needle, so that when sutures are tied, they will lift the tissues upward and forward toward under surface of the flap and pubic arch, and obliterate any rectocele present.

The flap-splitting method is the ideal operation in complete lacerations, by modifying the incision so as to form a rectal flap, also expose ends of sphincter ani so it may be approximated individually—then operation is completed as in an ordinary operation. If the operation is done properly a good perineal body is built up and a firm band of tissue may be felt across pelvic floor. The after pain is much less than in any operation where the sutures are introduced from the outside through skin and whole perineal body. The rectocele is easily corrected, and the mucous flap, if not completely obliterated in the stitching up of perineum, will soon disappear. The operation gives good deflection of fecal matter in rectum and preserves the whole of vaginal mucous membrane for elasticity in future labors.

## Figures and Tables

**Fig. 1. f1:**
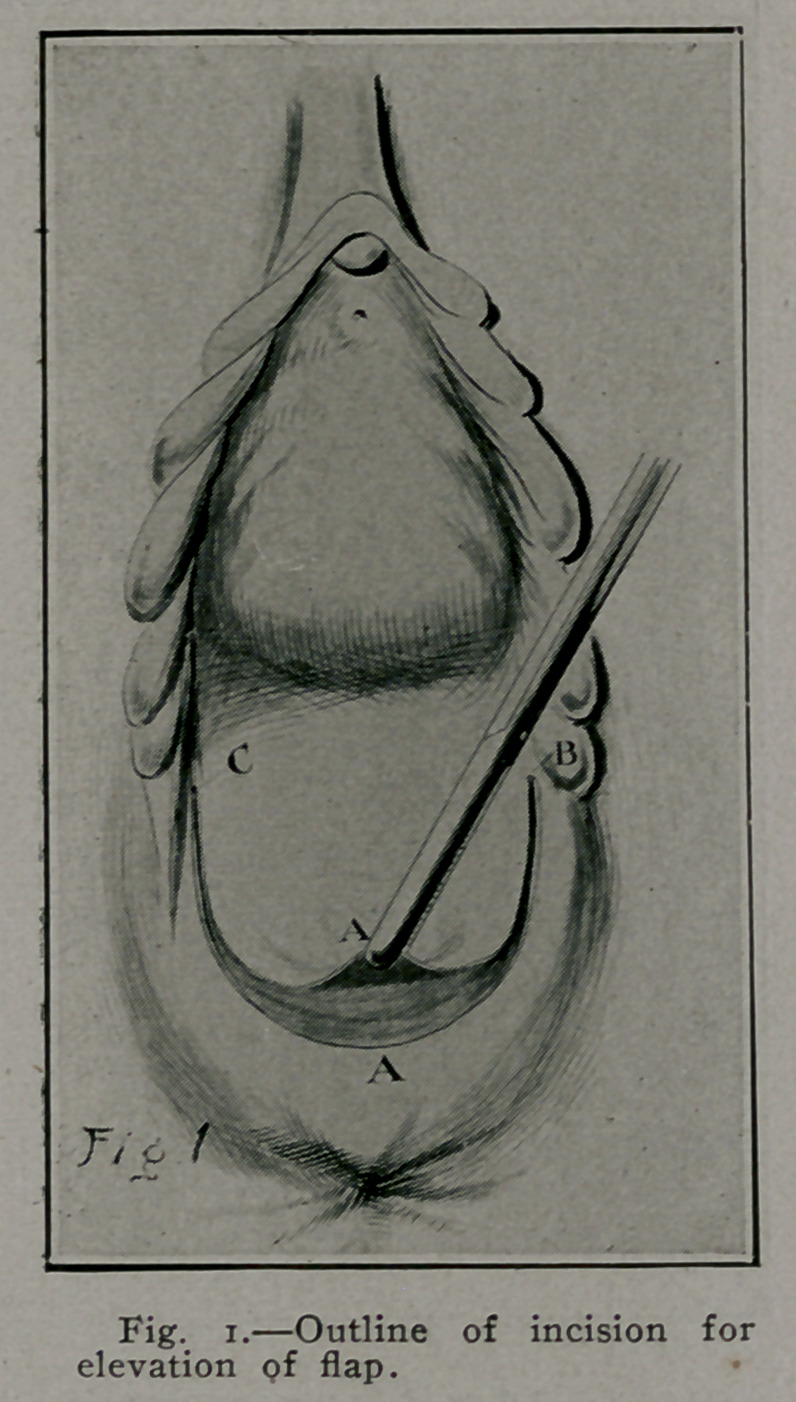


**Fig. 2. f2:**
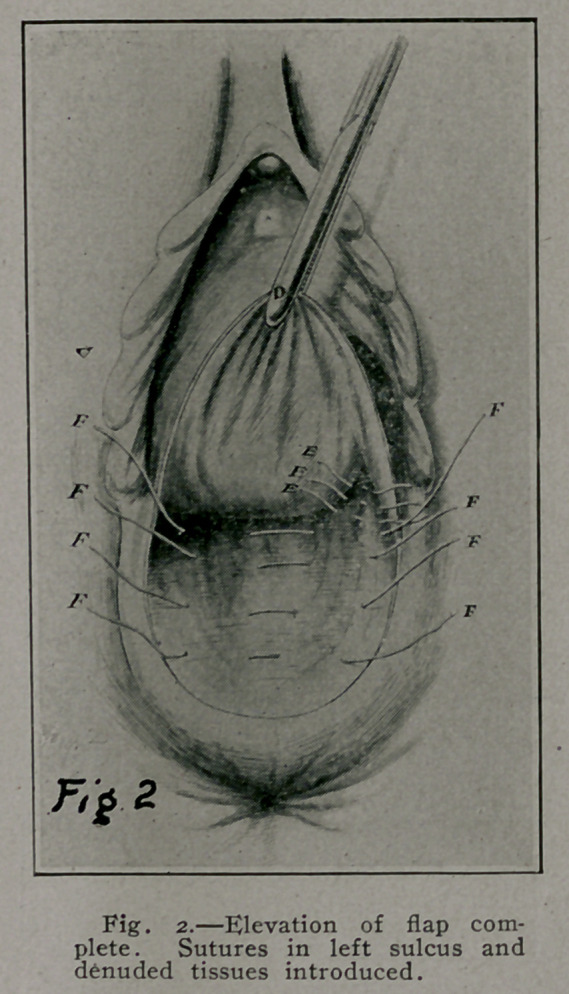


**Fig. 3. f3:**
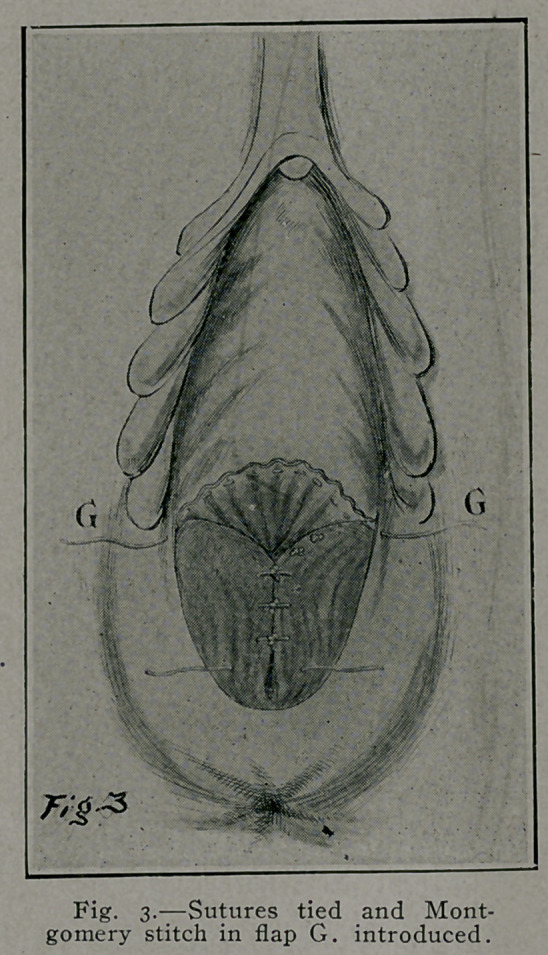


**Fig. 3. f4:**
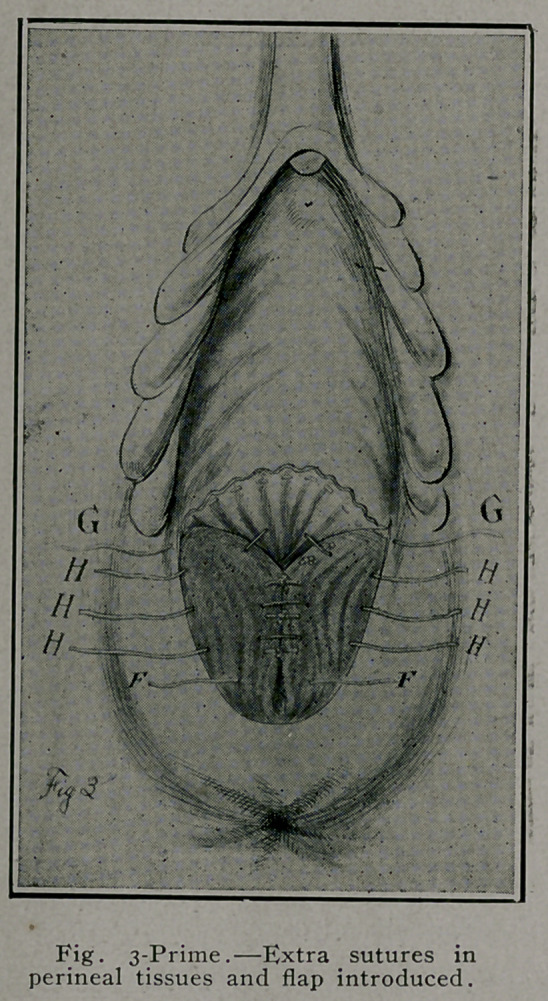


**Fig. 4. f5:**
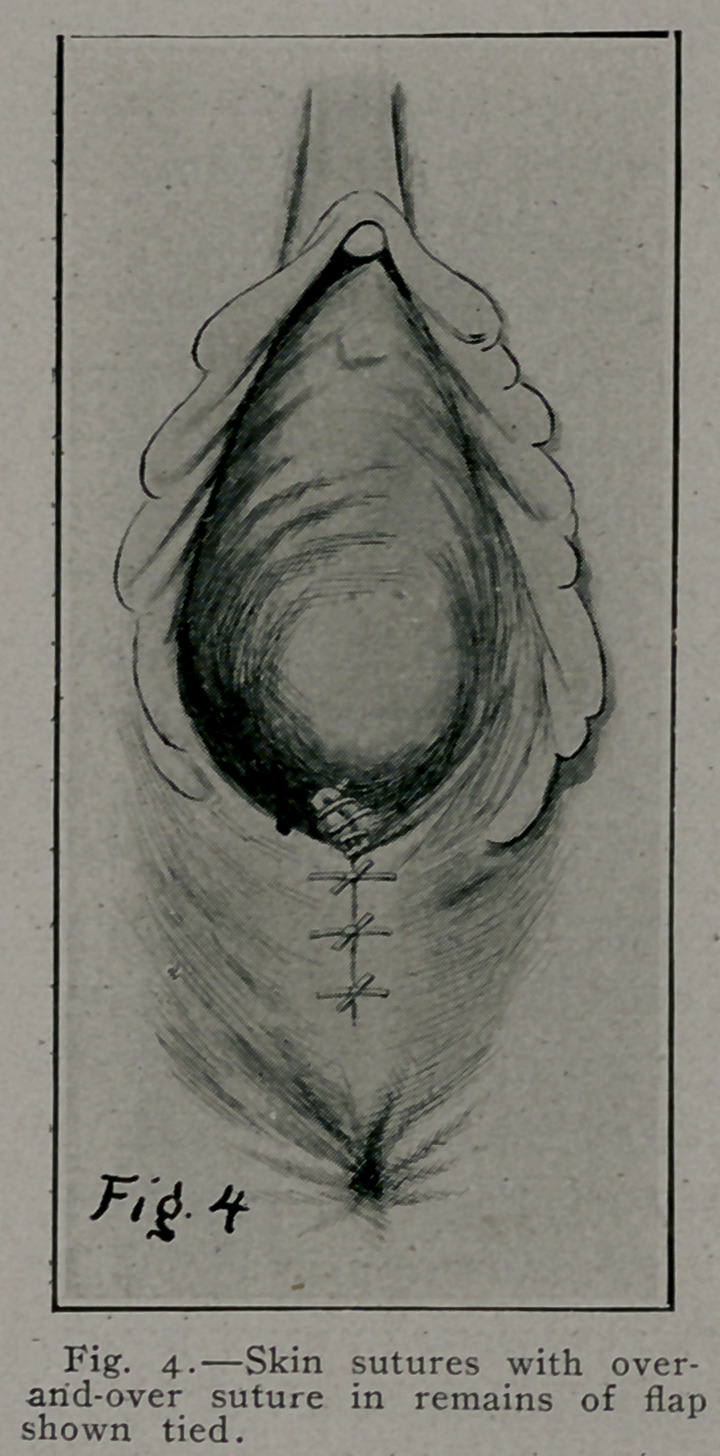


**Fig. 4. f6:**